# Supplementary material for: Wild-Grown and Cultivated Glechoma hederacea L.: Chemical Composition and Potential for Cultivation in Organic Farming Conditions
Source: Plants (Basel). 2022 Mar 18;11(6):819. doi: 10.3390/plants11060819 (PMC8949430; doi:10.3390/plants11060819)
Supplement: Supplementary file 1 [file plants-11-00819-s001.zip › plants-1647008-supplementary.pdf]

Supplementary Materials

Wild-grown and cultivated *Glechoma hederacea* L.: chemical composition and potential for cultivation in organic farming conditions

Inga Sile[1,2*], Valerija Krizhanovska[1,3], Ilva Nakurte[4], Ieva Mezaka[4], Laura Kalane[4], Jevgenijs Filipovs[4], Alekss Vecvanags[4], Osvalds Pugovics[1], Solveiga Grinberga[1], Maija Dambrova[1,3], Arta Kronberga[5]

[1]*Latvian Institute of Organic Synthesis, 21 Aizkraukles Str., LV-1006, Riga, Latvia*
[2]*Department of Applied Pharmacy, Riga Stradins University, 16 Dzirciema Str., Riga, LV-1007, Latvia*
[3]*Department of Pharmaceutical Chemistry, Riga Stradins University, 16 Dzirciema Str., Riga, LV-1007, Latvia*
[4]*Institute for Environmental Solutions, "Lidlauks", Priekuļi parish, Cēsis County, LV-4126, Latvia*
[5]*Field and Forest, SIA, 2 Izstades Str, Priekuli parish, Cēsis County, LV-4126, Latvia*

*Corresponding author e-mail: inga.sile@farm.osi.lv

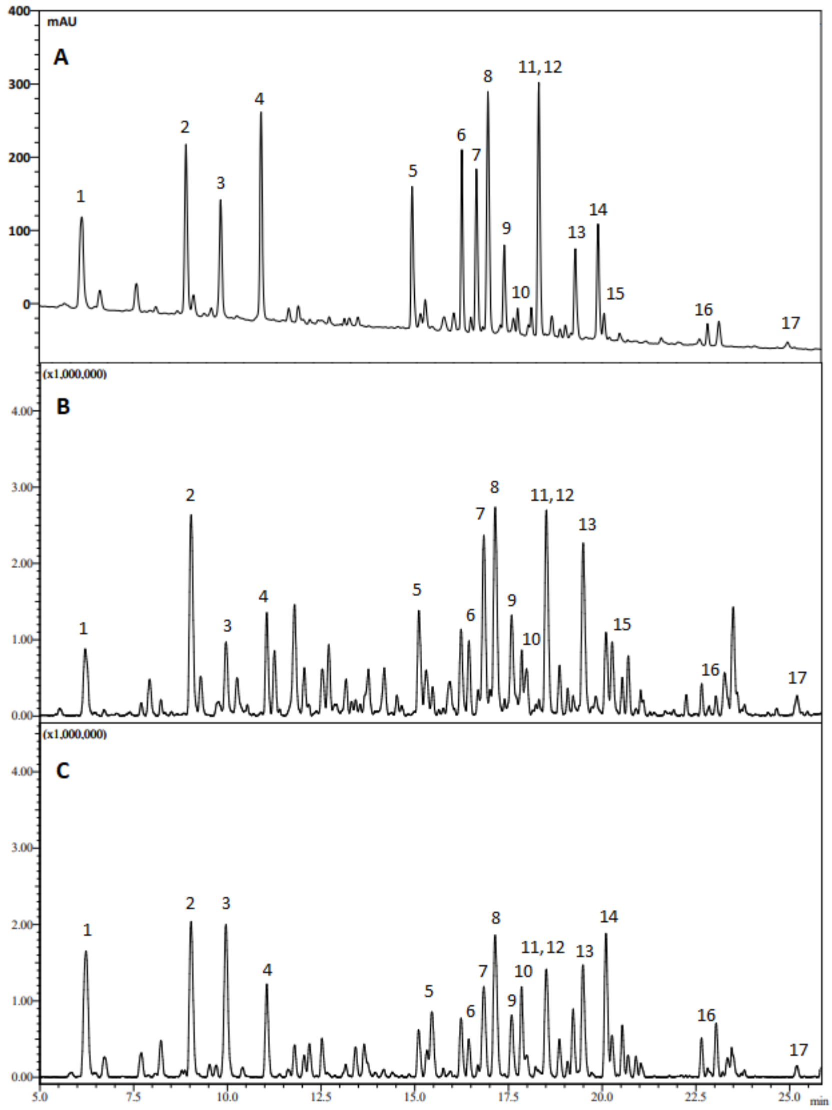

Figure S1. Representative chromatogram of *G. hederacea* 70% ethanol extract, (A) UHPLC-PDA (254 nm), (B) and (C) UHPLC-HRMS in positive and negative ionization mode, respectively.

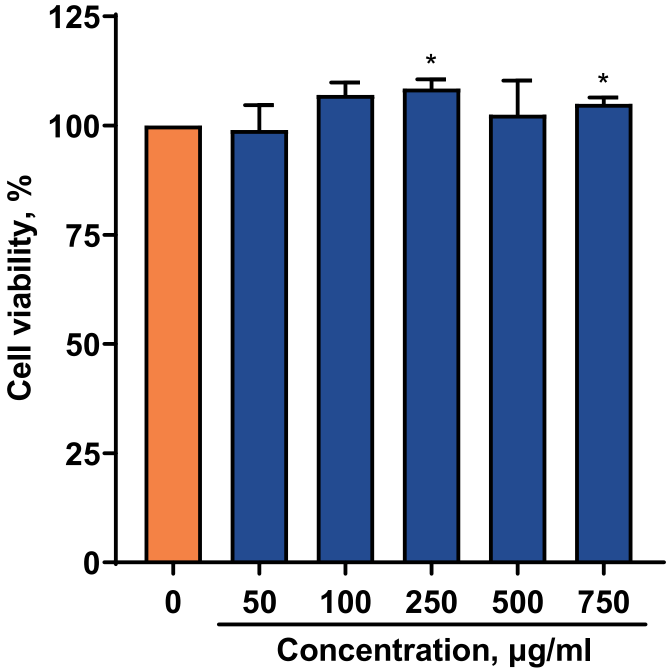

Figure S2. Effects of *G. hederacea* extract on bone marrow-derived macrophage viability measured by the MTT assay. Cell viability was tested after 24 h of incubation. Values are represented as the mean ± SD of three independent measurements in six parallels. * - significantly different from the untreated control (unpaired t-test, p<0.05).

Table S1. Volatile compound composition (%) of the essential oil of dry plants of *G. hederacea* extract harvested during 2019-2021

RI[a]	Compound[b]	Composition range Wild (Flowering)	Composition range Cultivated (Flowering and vegetative)
921	Cumene	0.9-10.2	0.5-14.2
929	1R-α-Pinene	n.d	n.d-0.5
962	Benzaldehyde	n.d	n.d-2.9
966	β-Thujene	n.d	n.d-0.4
980	1-Octen-3-ol	2.9-5.9	1.4-10.9
986	3-Octanone	n.d-0.8	n.d-1.3
991	β-Myrcene	0.8-4.7	1.0-5.7
994	3-Octanol	n.d	n.d-0.4
1028	β-Terpinene	n.d-0.6	n.d-1.5
1032	Eucalyptol	7.1-22.6	2.0-28.5
1038	cis-β-Ocimene	4.1-15.0	2.8-37.3
1049	trans-β-Ocimene	n.d	n.d-0.4
1104	Nonanal	n.d	n.d-0.6
1111	1-Octen-3-yl-acetate	n.d	n.d-0.5
1316	2-Acetyl-4-methylphenol	n.d	n.d-0.2
1338	δ-EIemene	n.d	n.d-0.8
1344	exo-2-Hydroxycineole acetate	n.d	n.d-0.2
1357	Eugenol	n.d	n.d-2.1
1376	Copaene	n.d	n.d-0.6
1384	β-Bourbonene	n.d-2.1	0.8-3.8
1391	β-Elemene	1.1-4.4	0.2-9.1

1411	α-Cedrene	n.d	n.d-0.1
1419	Caryophyllene	n.d-0.5	n.d-0.8
1432	β-Gurjunene	n.d	n.d-0.8
1433	γ-Elemene	n.d-1.1	n.d-1.2
1439	α-Guaiene	n.d	n.d-0.1
1454	Humulene	n.d-0.7	n.d-1.6
1475	4-epi-α-Acoradiene	n.d	n.d-0.3
1481	Germacrene D	15.0-34.1	12.6-34.5
1486	β-Eudesmene	n.d	n.d-0.4
1492	Valencene	n.d	n.d-4.1
1495	Bicyclogermacrene	1.5-2.2	0.4-4.0
1505	δ-Guaiene	2.5-4.0	n.d-8.5
1513	γ-Cadinene	n.d	n.d-0.2
1518	β-Cadinene	n.d-0.9	n.d-1.2
1549	Elemol	n.d	n.d-1.1
1557	Germacrene B	17.8-22.4	0.3-24.5
1573	1,5-Epoxysalvial-4(14)-ene	n.d	n.d-0.4
1574	Germacrene D-4-ol	n.d-0.6	0.5-2.2
1576	Spathulenol	n.d-0.6	0.3-2.9
1635	Patchoulane	n.d	n.d-0.4
1631	γ-Eudesmole	n.d-0.7	0.3-1.5
1638	Isospathulenol	n.d-0.5	0.4-0.8
1640	tau.-Cadinol	n.d	n.d-0.4
1642	Cubenol	n.d	n.d-0.4
1653	α-Cadinol	n.d-1.0	n.d-1.3
1655	Pogostole	n.d	n.d-0.6
1681	α-Santalol	n.d	n.d-0.3
1688	Eudesma-4(15),7-dien-1β -ol	n.d	n.d-0.3
1694	Eremophila-1,11-dien-9-one	n.d	n.d-0.4
1695	ent-Germacra-4(15),5,10(14)-trien-1β-ol	n.d	n.d-0.8
1700	α-trans-Bergamotenol	n.d	n.d-0.4
1744	α-Mintsulfide	n.d	n.d-0.2
1763	Lanceol, cis	n.d	n.d-0.6
1777	15-Hydroxy-α-muurolene	n.d-0.8	n.d-0.8
1826	8-Ketoylangenal	n.d	n.d-1.2
1844	Hexahydrofarnesyl acetone	n.d-0.6	n.d-1.1
1859	Ethanone, 1-[6-hydroxy-2-(1-methylethenyl)-5-benzofuranyl]-	n.d	n.d-0.6
1939	Verrucarol	n.d	n.d-1.4
2073	Thunbergol	n.d	n.d-0.8
2085	E-15-Heptadecenal	n.d	n.d-1.2
2098	Methyl linolenate	n.d	0.3-4.2
2114	Phytol	2.7-5.7	0.3-5.0
2153	Ethyl 9α-linolenate	n.d	n.d-1.7

[a] Retention indexes (RI) determined on HP-5MS capillary column
[b] Based on NIST (National Institute of Standards and Technology) MS search 2.2 library
n.d. – not detected

Table S2. Locations of the collected wild *G. hederacea* accessions

Accession denomination	Municipality	Latitude	Longitude	Elevation
GH01	Ainaži	57°52'12.2"N	24°38'15.7"E	48 m
GH02	Viļaka	57°10'47.9"N	27°40'51.2"E	94 m
GH03	Sigulda	57°09'14.2"N	24°53'54.5"E	98 m

Table S3. MRM parameters applied for the analysis of flavonoids in *G. hederacea* extract

Compound	MS/MS	Cone, V	Collision energy, eV
Apigenin 7-O-glucoside	433 > 271	30	15
Kaempferol 3-O-rutinoside	595 > 287	30	15
Rutin	611 > 303	30	20
Hyperoside	465 > 303	30	15
Luteolin 7-O-glucoside	449 > 287	30	15
Apigenin	271 > 111, 271 > 163	30	30
Luteolin	287 > 121, 287 > 165	25	30

Table S4. MRM parameters applied for the analysis of phenolic acids in *G. hederacea* extract

Compound	MS/MS	Cone, V	Collision energy, eV
Chlorogenic acid	353 > 191	20	15
Rosmarinic acid	359 > 161	20	15
Caffeic acid	179 > 135	25	15
